# Translocation of Phytoliths Within Natural Soil Profiles in Northeast China

**DOI:** 10.3389/fpls.2019.01254

**Published:** 2019-10-17

**Authors:** Lidan Liu, Dehui Li, Dongmei Jie, Hongyan Liu, Guizai Gao, Nannan Li

**Affiliations:** ^1^College of Resources and Environmental Science, Hunan Normal University, Changsha, China; ^2^Institute for Peat and Mire Research, State Environmental Protection Key Laboratory of Wetland Ecology and Vegetation Restoration, Northeast Normal University, Changchun, China; ^3^Key Laboratory of Geographical Processes and Ecological Security in Changbai Mountains, Ministry of Education, Changchun, China; ^4^Key Laboratory of Vegetation Ecology, Ministry of Education, Changchun, China; ^5^Resources Environment & Tourism, Anyang Normal University, Anyang, China

**Keywords:** phytolith, transport characteristics, paleovegetation, climatic proxy, Northeast China

## Abstract

Phytoliths are a reliable paleovegetation proxy and have made an important contribution to paleoclimatic studies. However, little is known about the depositional processes affecting soil phytoliths, which limits their use for paleoclimate and paleovegetation reconstructions. Here, we present the results of a study of the vertical translocation characteristics of phytoliths in 40 natural soil profiles in Northeast China. The results show that phytolith concentration decreases within the humic horizon of the soil profiles and that ∼22% of the phytoliths are translocated below the surface of the studied soils. In addition, we find that the translocation rate of phytoliths varies markedly with phytolith type and that phytolith size and aspect ratio also have a significant effect. Phytoliths with length >30 μm and with aspect ratio >2 and those with length <20 μm and aspect ratio <2 are preferentially translocated compared to those with length >25 μm and aspect ratio <2. Our results demonstrate that differential translocation of phytoliths within soil profiles should be considered when using soil phytoliths for paleoclimate and paleovegetation reconstruction.

## Introduction

Phytoliths are microscopic silica bodies that precipitate in or among cells of living plant tissues. Owing to their abundance and environmental sensitivity, the use of phytoliths as an environmental indicator has received increasing attention. Specifically, phytolith analysis has been widely used in paleovegetation reconstructions, such as monitoring shifts in forest–grassland boundaries, vegetation succession, and changes in alpine timberlines (Barboni et al., 2007; Ákos, 2013; Coe et al., 2013; Dickau et al., 2013; Song et al., 2016; Li et al., 2017; Novello et al., 2017). However, it has been observed that soil phytoliths are subject to preservation bias, and they can be dissolved from archaeological and sedimentary records under alkaline conditions, or due to mechanical abrasion, and partially dissolved phytoliths will more easily break into fragments (Fraysse et al., 2009; Tsartsidou et al., 2009; Cabanes et al., 2011; Novello et al., 2012; Albert et al., 2015; Prentice and Webb, 2016). In addition, under the influences of wind, surface runoff, and human activity, soil phytoliths can be horizontally migrated (Wallis, 2001; Farmer et al., 2005; Esteban et al., 2017; Bremond et al., 2017), and phytoliths maybe also translocated beneath the soil surface due to various taphonomic events (Osterrieth et al., 2009; Golyeva and Svirida, 2017). Such dissolution and translocation effects can result in the misinterpretation of poorly preserved phytolith assemblages, which reduces their reliability for palaeovegetation and paleoclimatic reconstructions. Therefore, when using soil phytoliths for paleoclimate and paleovegetation reconstructions, the effects of these processes must be considered, and a first step is to improve our understanding of modern processes affecting phytoliths by conducting a study of their translocation within soil profiles.

To date, research on the vertical translocation of soil phytoliths has been conducted in several geographical regions (Borba-Roschel et al., 2006; Bradford et al., 2006; Cabanes et al., 2012; Li et al., 2012; Boixadera et al., 2016; Inoue et al., 2016). These studies have mainly focused on characterizing changes in phytolith assemblages with soil depth, and the results indicate that phytolith quantity decreases with depth, principally within the surface layer of the soil profile (Bradford et al., 2006; Li et al., 2012). However, although the phenomenon of the vertical translocation of phytoliths can be found in undisturbed soils (Bradford et al., 2006), there are only a few studies about their translocation rates in natural soils. Experiments have been conducted using, for example, irrigation (a fluorescent labeling technique), in which phytoliths are added to phytolith-free sandy sediment or other soil types (Fishkis et al., 2009; Fishkis et al., 2010a). Changes in phytolith concentration with depth are then measured to determine the translocation rates of specific phytolith types (Fishkis et al., 2009; Fishkis et al., 2010a; Fishkis et al., 2010b). However, this experimental approach may overlook the complexity of factors influencing the vertical translocation of soil phytoliths under natural conditions, which results in an incomplete understanding of the processes involved. In addition, soil phytoliths are contributed by a wide variety of plant species, and even by mixtures of herbaceous plants and trees, but phytolith morphotypes studied so far are insufficient to be fully representative of soil phytoliths in different environments (Piperno, 2006). Moreover, most previous studies have focused on changes in phytolith morphologies with depth and sampling interval and have rarely considered the influence of soil formation on phytolith translocation.

Here, we present the results of a study of soil phytolith assemblages in 40 natural soil profiles in Northeast China and analyze the results from the perspectives of soil formation and soil horizonation. Our main aim is to investigate the rate of phytolith translocation within natural soils and the degree to which phytolith translocation depends on phytolith morphology. Our results potentially provide a basic scientific reference for the preservation characteristics of soil phytoliths, and they may help improve the reliability of phytolith-based paleoclimate and paleovegetation reconstructions in the temperate zone.

## Study Area

**Figure 1:**
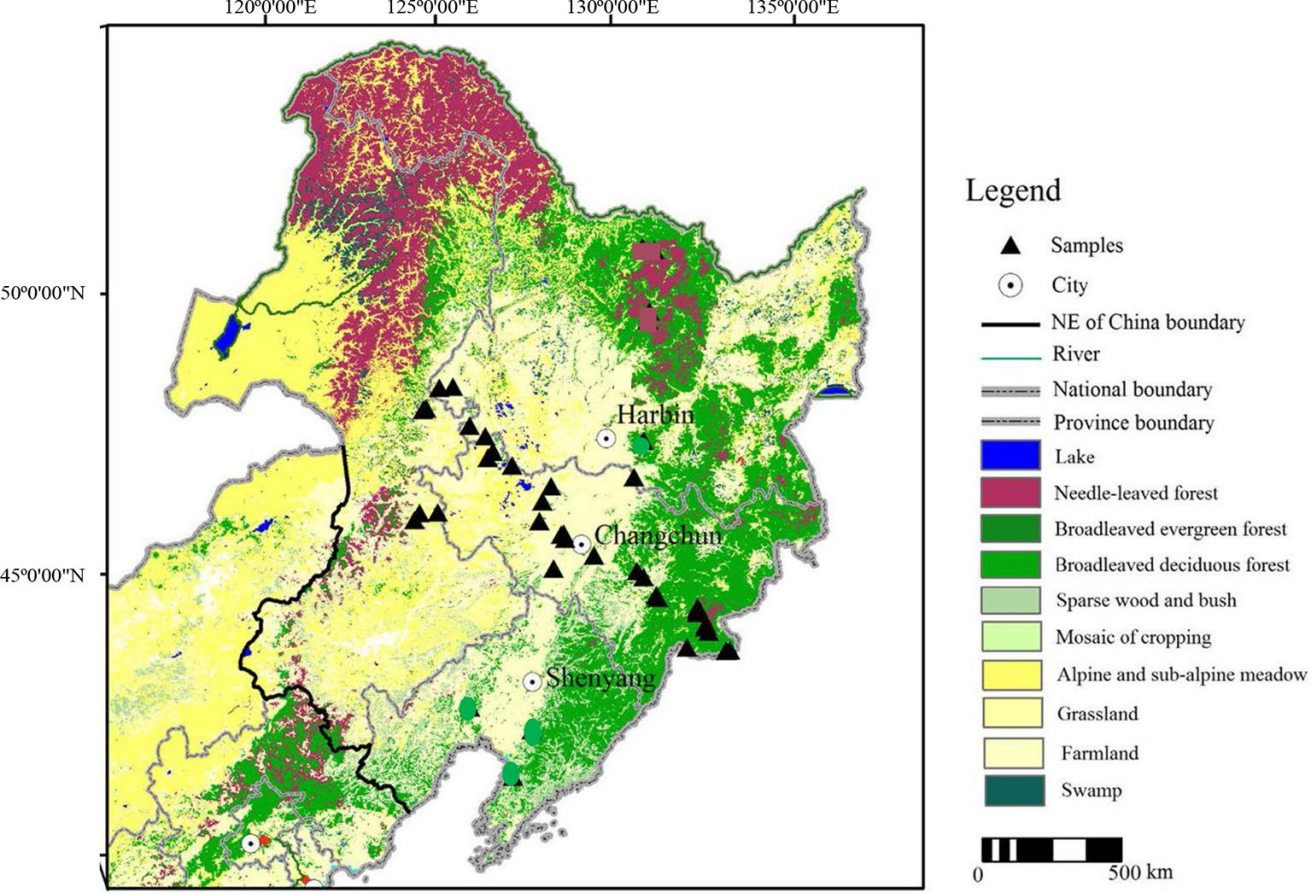
Location of sampling sites in Northeast China.

The study area in Jilin province of Northeast China is located at 39°40ʹN–53°30ʹN, 115°05ʹE–135°02ʹE (**Figure 1**) (Ma et al., 2007). The modern climate of the area is influenced by the East Asian monsoon, which has four distinct seasons. NE China also exhibits a large variety of soil types along with the vegetation changes, although they are all characterized by a high organic matter content. The vegetation zones within the study area exhibit a northeast–southwest (NE–SW) distribution, reflecting the orientation of thrust faults. In the Daxing’anling Mountains region, in the western part of NE China, which belongs to the cold temperate zone, its regional average annual temperature is −2.8°C, and average annual precipitation is 746 mm; coniferous forest is widely distributed, with *Larix gmelinii* as the dominant species; and Brown coniferous forest soils dominate in this zone. In the Changbai Mountains region, in the eastern part of NE China, which belongs to the temperate zone, its regional average annual temperature ranges from 2 to 6°C, and average annual precipitation ranges from 400 to 700 mm (Li et al., 2001); the natural vegetation is typically mixed coniferous-broadleaved forest, characterized by *Pinus koraiensis* and *Betula costata*; and dark brown soils and black soils mainly occur in this region. Songnen Plain, in the western part of NE China, belongs to the temperate zone, situated along the eastern margin of the temperate steppe in North China; its regional average annual temperature ranges from 3.5 to 5.0°C, and the average annual precipitation ranges from 360 to 480 mm (70% of the region’s precipitation falls in summer) (Li et al., 2017); locally, the vegetation changes to forest grassland, alternatively called meadow grassland, dominated for example by *Leymus chinensis* and *Stipa baicalensis*, with occasional trees such as *Populus davidiana* and *Ulmus pumila*; Chernozems and Dark brown soils mainly occur in this region. In addition, there are also several intrazonal soil types (e.g., albic, meadow, and peaty soils), which occasionally occur locally (Guo et al., 2008; Zhao et al., 2011).

## Materials and Methods

### Sample Collection

We collected samples from various soil types (dark brown soil, chernozem, chestnut soil, black soil, alluvial soil, and albic soil) and corresponding topsoil samples from 40 sampling sites in NE China (**Figure 1**). Forty soil profiles were collected based on soil horizonation, and as far as possible, we sampled all the diagnostic horizons within each profile. Forty topsoil samples were also collected from the uppermost 2–3 cm of surface soil, excluding the surface litter layer. Vegetation and soil profile information for these samples are listed in **Table 1**.

**Table 1:** Soil type, horizonation and vegetation community type of the sampling sites in Northeast China.

Number	Sampling site	Longitude(E)	Latitude(N)	Soil type	Horizonation	Vegetation community
1	Fusong(1)[FS(1)]	127°41′13.5″	41°52′7.6″	Dark brown soil	A,E,B	*Acer triflorum–Urtica laetevirens *community
2	Fusong(2)[FS(2)]	127°40′45.1″	41°58′18.7″	Dark brown soil	A,E	*Abies nephrolepis–Carex siderosticta* community
3	Fusong(3)[FS(3)]	127°36′0.6″	42°20′14.3″	Dark brown soil	A,E	*Acer mono–Urtica laetevirens* community
4	Fusong(4)[FS(4)]	127°23′29.7″	42°16′59.3″	Dark brown soil	A,E	*Acer mono–Carex siderosticta* community
5	Fusong(5)[FS(5)]	127°37′52.9″	41°57′42.5″	Alluvial soil	A,B,C	*Juncus effusus* community
6	Fusong(6)[FS(6)]	127°34′30.5″	42°19′41.8″	Alluvial soil	A,C	*Hippochaetehyemale* community
7	Fusong(7)[FS(7)]	127°31′42.9″	42°14′39.6″	Albic soil	A,E,B	*Pinnus koraiensis–Carex* community
8	Hudian(1)[HD(1)]	126°42′32.9″	42°44′58.9″	Dark brown soil	A,E,C,R	*Juglans mandshurica–Setaria viridis* community
9	Hudian(2)[HD(2)]	126°31′52.9″	43°07′35.3″	Dark brown soil	A,B,C,R	*Quercus mongolica–Carex siderosticta* community
10	Hudian(3)[HD(3)]	126°44′43.1″	42°42′13.2″	Albic soil	A,E,C	–
11	Changbai(1)[CB(1)]	128°02′41.5″	41°26′13.4″	Dark brown soil	A,E,C	*Fraxinus mandshurica–Carex rigescens* community
12	Changbai(2)[CB(2)]	127°04′33.9″	41°42′09.4″	Albic soil	A,E,B	–
13	Changbai(3)[CB(3)]	128°09′20.4″	41°23′23.6″	Alluvial soil	A,C	*Calamagrostisepigejos* community
14	Shuangyang(1)[SY(1)]	125°30′8.3″	43°44′18.3″	Dark brown soil	A,E,C	–
15	Shuangyang(2)[SY(2)]	124°27′18.2″	43°41′23.7″	Dark brown soil	A,E,C	–
16	Shuangyang(3)[SY(3)]	125°36′06.4″	43°32′09.0″	Black soil	A,B	–
17	Shuangyang(4)[SY(4)]	125°44′51.3″	43°23′49.7″	Albic soil	A,E,C	–
18	Changchun(1)[CC(1)]	125°3′27.8″	43°59′15″	Chernozem	A,B,C	–
19	Nong’an(1)[NA(1)]	124°41′11″	44°22′31.2″	Chernozem	A,B,C	*Leymus chinensis* community
20	Dehui(1)[DH(1)]	125°26′27.5″	44°0′33.4″	Black soil	A,B	–
21	Dehui(2)[DH(2)]	125°40′26.8″	44°16′19.0″	Black soil	A,B	–
22	Changling(1)[CL(1)]	124°31′18.4″	44°26′9.6″	Chernozem	A,B,C	*Leymus chinensis* community
23	Changling(2)[CL(2)]	124°25′20.8″	44°29′30.5″	Chernozem	A,B	–
24	Qianguo(1)[QG(1)]	124°31′25.6″	45°25′27.3″	Chernozem	A,B,C	*Stipa capillata* community
25	Qianguo(2)[QG(2)]	124°21′22.7″	45°24′2.5″	Chernozem	A,B	–
26	Baicheng(1)[BC(1)]	123°2′23.5″	44°57′40.7″	Chernozem	A,B,C	–
27	Baicheng(2)[BC(2)]	122°49′38.3″	45°15′4.6″	Chernozem	A,B,C	–
28	Da’an(1)[DA(1)]	124°16′12.8″	45°32′4.9″	Chernozem	A,B,C	*Leymus chinensis* community
29	Da’an(2)[DA(2)]	124°14′39.2″	45°32′30.4″	Chernozem	A,B,C	*Dactylocteniumwilld* community
30	Zhenlai(1)[ZL(1)]	123°42′53.6″	45°57′51.1″	Chernozem	A,B,C	*Stipa capillata* community
31	Zhenlai(2)[ZL(2)]	123°30′59.1″	45°54′24.4″	Chernozem	A,B,C	–
32	Tailai(1)[TL(1)]	123°37′22.1″	46°17′7.1″	Chernozem	A,B,C	*Leymus chinensis* community
33	Tailai(2)[TL(2)]	123°40′49.5″	46°54′23.4″	Alluvial soil	A,C	*Poa* community
34	Longjiang(1)[LJ(1)]	122°55′56.1″	47°14′40.3″	Dark brown soil	A,C	–
35	Longjiang(2)[LJ(2)]	122°44′21.6″	47°18′36.8″	Dark brown soil	A,E	–
36	Longjiang(3)[LJ(3)]	123°0′7″	47°18′51.5″	Chernozem	A,B	*Leymus chinensis* community
37	Zhalaite(1)[ZLT(1)]	122°28′31.1″	47°8′24.3″	Dark brown soil	A,C	–
38	Zhalaite(2)[ZLT(2)]	122°7′22.5″	46°58′39.4″	Dark brown soil	A,C	–
39	Zhalaite(3)[ZLT(3)]	122°28′5.4″	47°2′24.3″	Black soil	A,B	–
40	Neimeng(1)[NM(1)]	121°20′17.0″	45°06′42.9″	Chestnut soil	A,E,B,R	*Setaria viridis* community

A, humus horizon (A horizon); B, illuvial horizon (B horizon); C, parent material horizon (C horizon); E, eluvial horizon (E horizon); R, bed rock horizon (R horizon).

### Phytolith Extraction Methods

Phytoliths were extracted from topsoil and soil profile samples using the wet ashing method (Li et al., 2017). The soil samples were air dried overnight at 80°C and then pulverized into a powder, and 5 g of sieved soil was weighed and added to a 50-ml centrifuge tube. To remove carbonates, 10% HCl was added, and the samples were stirred regularly until the reaction ceased. Distilled water was then added, and the mixture was centrifuged three times at 2,000 rpm for 20 min. To remove organic matter, concentrated HNO_3_ was added and the samples heated in a water bath at 90°C until the reaction subsided. Distilled water was then added, and the samples were centrifuged at 2,000 rpm for 20 min. Phytoliths were then extracted by floatation using a ZnBr_2_ solution with a specific gravity of 2.38, together with centrifugation; the supernatant was collected and washed with distilled water. Next, a known number of *Lycopodium* spores was added to another centrifuge tube and mixed with 10% HCl, which was then added to the abovementioned supernatant, and the mixture was centrifuged twice. Absolute ethanol was then added to the centrifuge tube, and the mixture was centrifuged at 2,000 rpm for 20 min. Finally, one to three drops of the suspension were placed on a glass microscope slide, which was heated over a spirit lamp until all the ethanol was evaporated. Canada balsam oil (one to two drops) was added and a cover slip placed on top. Observations and identification were performed with an Olympus microscope at a magnification of ×600. At least 300 phytolith grains were counted for each sample.

In addition, a phytolith concentration is the amount of phytoliths per gram of dry soil, and the formula of phytolith concentration:

$$w=\frac{n\times M}{N\times m}$$

In the formula, *n* represents the number of the phytolith in each slide, *N* represents the number of lycopodium spores in each slide, *M* represents the number of lycopodium spore in a slice of lycopodium spores, *m* represents the weight of each experimental samples (g), and finally calculate the phytolith concentrations *w* (10^3^ particles/g).

The physical composition was tested using a laser diffraction particle size analyzer (Microtrac S3500, Montgomeryville, Pennsylvania, USA), which can measure particle sizes from 0.02 to 2,800.00 μm. More detailed information about the procedure for determining the physical composition is given in Ahmed et al. (2016) and Ordóñez et al. (2016).

## Results

### Distribution of Phytoliths Within Natural Soil Profiles

**Figure 2:**
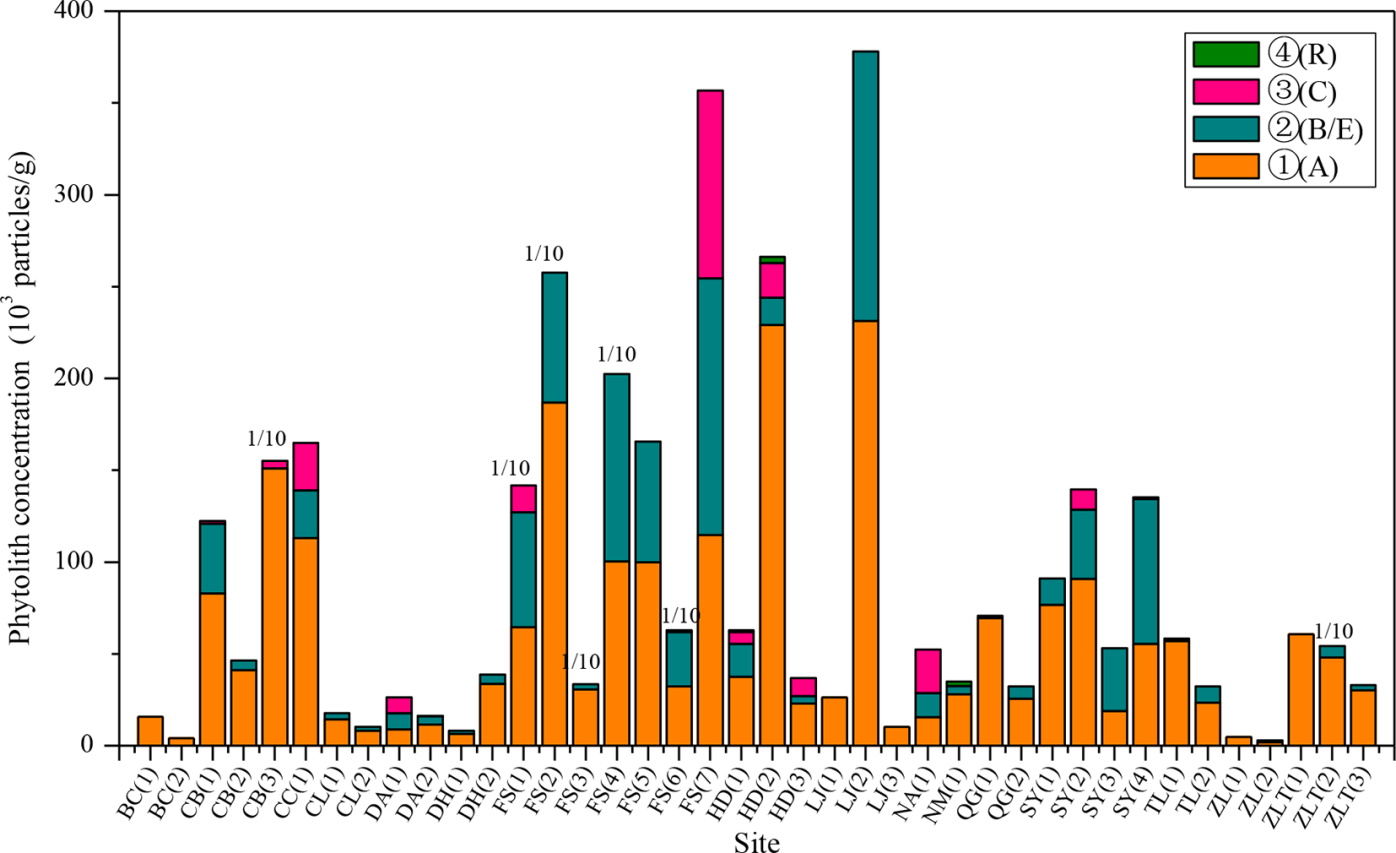
Distribution characteristics of phytolith concentration within the horizons of the soil profiles from different sampling sites in Northeast China. A, B, C, E, and R are soil horizons. 1/10: the concentration of the sampling site is the one tenth of its actual concentration.

The phytolith distributions in relation to the horizonation of the studied soils are illustrated in **Figure 2** and **Table 2**. In most of the soil profiles, the depth distribution of phytoliths exhibits a consistent pattern. Phytolith concentration decreases systematically with depth, from the humic horizon (Ahorizon) to the parent material (Chorizon). However, there are several exceptions: at some sites, for example, the phytolith distribution exhibits the opposite distribution, e.g., in the profiles from Shuangyang (3), Shuangyang (4), and Fusong (6). In general, however, the depth distribution of phytoliths in the profiles exhibits a similar pattern, with highest concentrations occurring in the humic horizon.

**Table 2:** Phytolith concentration within the horizons of the soil profiles from different sampling sites in Northeast China.

Number	Site	Soil horizon				Number	Site	Soil horizon			
		①(A)	②(B/E)	③(C)	④(R)			①(A)	②(B/E)	③(C)	④(R)
1	BC(1)	15.81	0.00	–	–	21	HD(2)	229.05	14.93	18.87	3.23
2	BC(2)	4.00	0.00	0.00	–	22	HD(3)	22.94	3.99	9.88	–
3	CB(1)	82.99	37.87	1.59	–	23	LJ(1)	26.28	0.00	–	–
4	CB(2)	41.02	5.25	–	–	24	LJ(2)	231.32	146.69	–	–
5	CB(3)	151.02	0.00	4.09	–	25	LJ(3)	10.24	0.00	–	–
6	CC(1)	113.06	26.10	25.67	–	26	NA(1)	15.52	13.08	23.67	–
7	CL(1)	14.40	3.37	0.00	–	27	NM(1)	27.88	4.69	0.00	2.22
8	CL(2)	8.03	2.13	–	–	28	QG(1)	69.49	1.24	0.00	–
9	DA(1)	8.95	8.63	8.67	–	29	QG(2)	25.68	6.51	–	–
10	DA(2)	11.57	4.48	0.31	–	30	SY(1)	76.63	14.34	0.00	–
11	DH(1)	6.50	1.72	–	–	31	SY(2)	90.70	37.93	11.00	–
12	DH(2)	33.78	4.88	–	–	32	SY(3)	18.98	34.06	–	–
13	FS(1)	644.90	626.53	146.07	–	33	SY(4)	55.45	78.92	0.94	–
14	FS(2)	1,868.57	707.37	–	–	34	TL(1)	56.79	1.16	0.29	–
15	FS(3)	305.92	27.99	–	–	35	TL(2)	23.49	8.87	–	–
16	FS(4)	1,003.18	1,019.72	–	–	36	ZL(1)	4.72	0.00	0.00	–
17	FS(5)	99.80	65.74	–	–	37	ZL(2)	1.86	0.23	0.66	–
18	FS(6)	321.53	297.65	8.38	–	38	ZLT(1)	60.72	0.00	–	–
19	FS(7)	114.65	139.75	102.46	–	39	ZLT(2)	479.66	62.71	–	–
20	HD(1)	37.57	17.91	6.43	0.83	40	ZLT(3)	30.18	2.78	–	–

**Figure 3:**
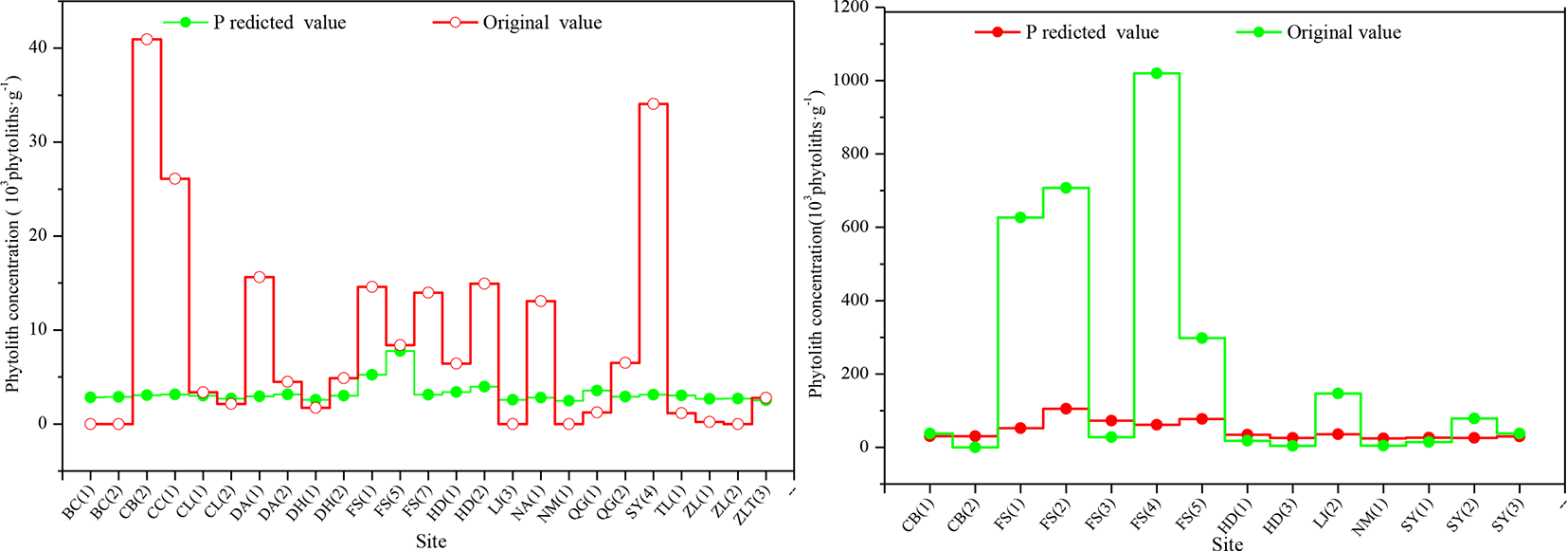
Comparison of the original and predicted phytolith concentrations in the B (left) and C (right) horizons of soil profiles in Northeast China. The estimated concentration is based on a linear regression model.

To confirm the phytolith content of different soil horizons derived from the aboveground vegetation, we studied the relationship between phytolith concentration and soil organic matter content and found that there was a closely linear relationship between phytolith concentration and soil organic matter content, which was also found in previous studies (Zhang et al., 2011). Thus, we used the linear regression equation [*Y* = 0.5098 + 2.7971*x*, where *Y* = phytolith concentration and *x* = soil organic matter content (*R* = 0.673, *F* = 123.448, *p* = 0.000)] to estimate the phytolith concentrations of the soil profiles derived from the aboveground vegetation and compared the results with the original phytolith concentrations (**Figure 3**). Except for a few sites, the predicted phytolith concentrations of the illuvial (B) and eluvial (E) horizons of the soil profiles are all lower than the original values. On average, the original values of phytolith concentration of the B and E horizons of the soil profiles are six and four times greater than the predicted values, respectively.

**Figure 4:**
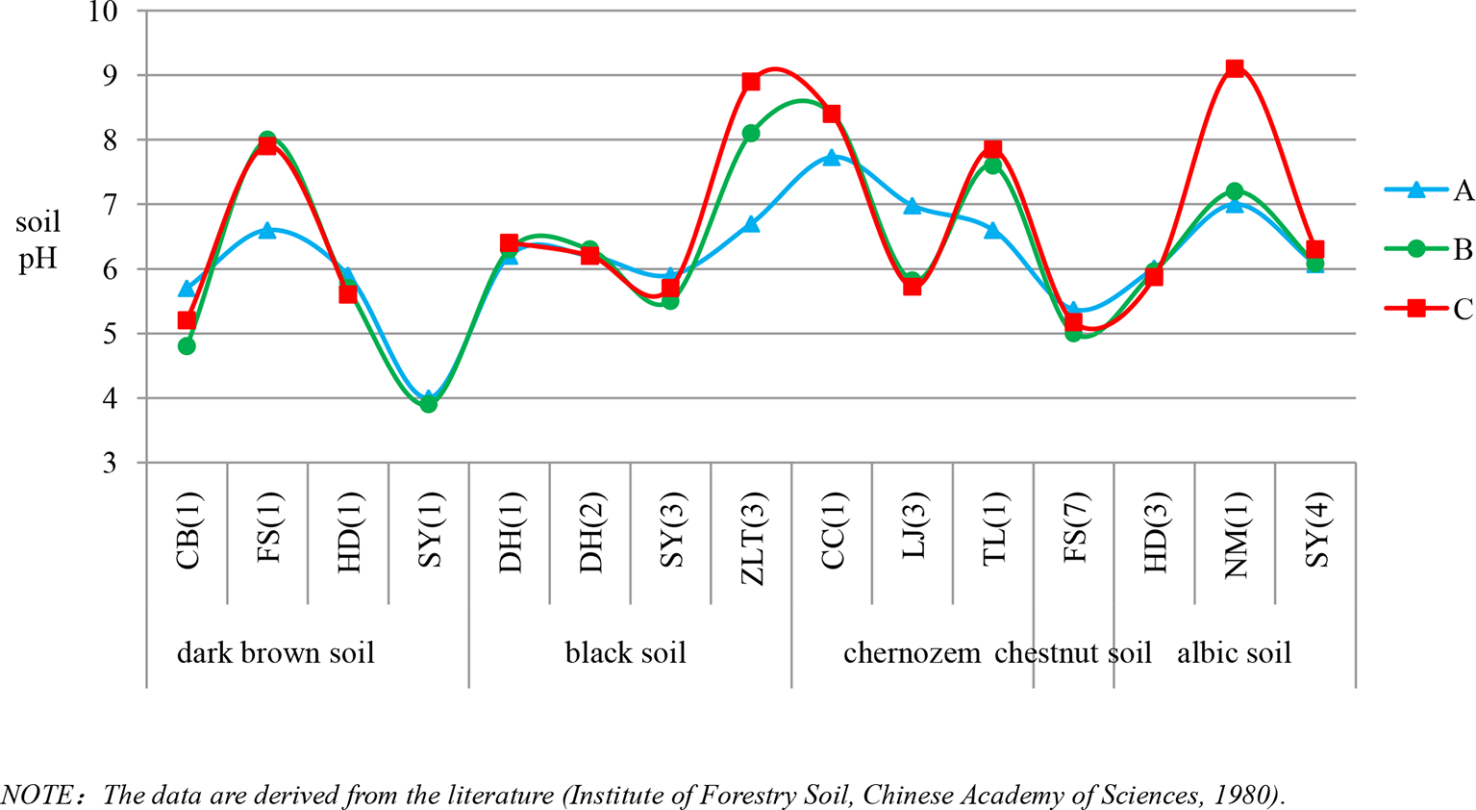
Variation of pH within the horizons of different soil types in Northeast China. A, B and C are soil horizons.

**Figure 5:**
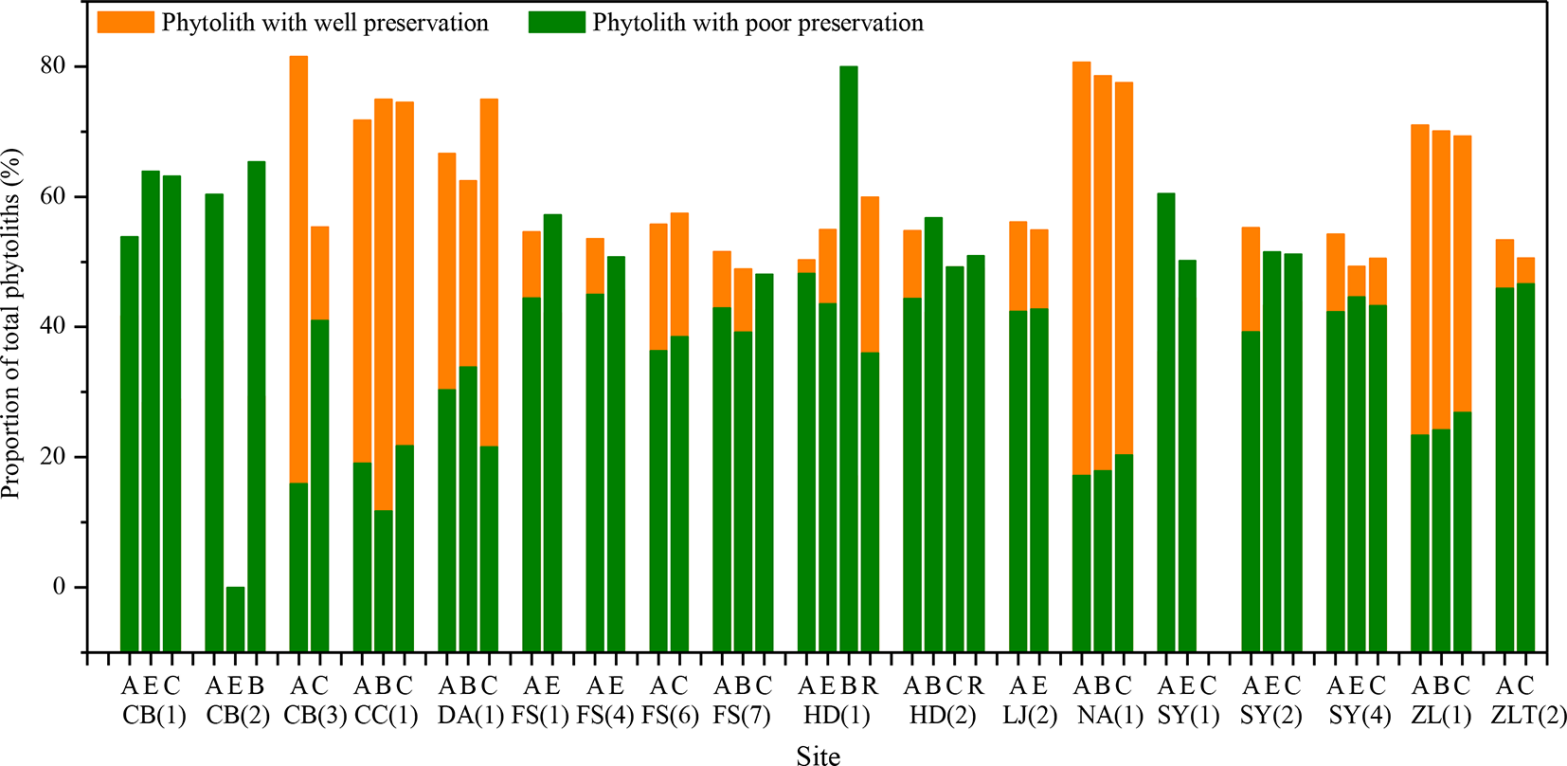
Distribution of well- and poorly-preserved phytoliths within different soil horizons in the studied soil profiles in Northeast China. A, B, C, E, and R are soil horizons.

Based on findings of soil phytolith preservation, we also examined differences in the content of poorly preserved (short-cell phytoliths and Tabular) and well-preserved phytoliths (lanceolate, elongate, blocky, and bulliform) (Albert et al., 2006; Cabanes et al., 2011) within different horizons of the soil profiles. In ∼32% of the sample sites, the depth distribution of poorly preserved phytoliths exhibits a similar pattern, with the highest content in the lower layer (**Figure 4**). Combined with **Figure 5**, specifically, in the lower soil layers, soil pH is high, whereas the content of poorly preserved phytoliths is also high.

**Figure 6:**
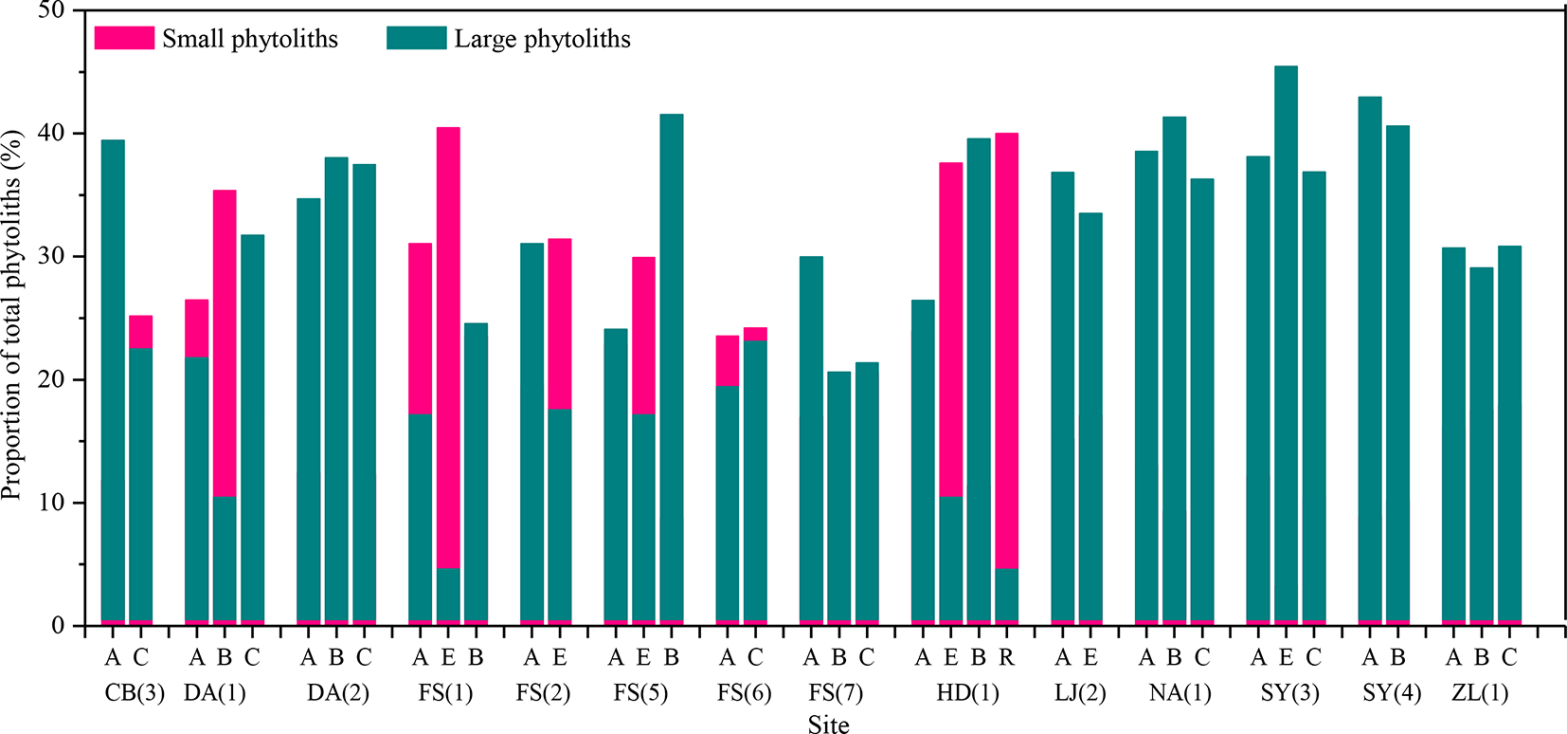
Variation of the proportions of large and small phytoliths between the horizons of soil profiles in Northeast China. A, B, C, E, and R are soil horizons.

To further assess vertical translocation of phytoliths within natural soil profiles, the proportions of large and small phytoliths are calculated. The small phytoliths mainly include short-cell phytolits (e.g., saddle, rondel, bilobate, and trapeziform sinuate) (Gu et al., 2013), whereas large phytoliths contain lanceolate, elongate, tabular, blocky, and bulliform. Subsequently, a comparison of the proportions of large and small phytoliths with depth was made (**Figure 6**). In the present dataset, the size distribution of phytoliths with depth within the profiles is consistent with pollen. In ∼29% of the sample sites, the content of small phytoliths increases with depth, whereas the content of large phytoliths decreases. Thus, we conclude that, in natural soil profiles, small phytoliths prefer to distribute in the lower layers.

### Vertical Translocation Rates of Phytoliths in Natural Soil Profiles

#### Total Vertical Translocation Rate of Phytoliths

In the studied soil profiles, the original phytolith concentration of the illuvial horizon (Bhorizon) and eluvial horizon (Ehorizon) is respectively six and four times greater than the values estimated by the linear regression model (see Section “Distribution of Phytoliths Within Natural Soil Profiles”). Here, the predicted values of phytolith concentration of the Bhorizon and Ehorizon are regarded as the actual phytolith concentration derived from the aboveground vegetation. In addition, using the above rations (i.e., six and four), we recalculated the phytolith concentration of the Bhorizon and Ehorizon of the soil profiles caused by phytolith translocation. Finally, the recalculated phytolith concentration of the B, E, and C (or R) horizons may be the result of phytolith transport from the upper layers of the soil profile. To determine the phytolith translocation rate in natural soils, we defined various phytolith translocation indices, including the total translocation rate (*T*), translocation rate of the Chorizon (*CT*), and the relative translocation rate of the Chorizon (*CT*′). The formulae are listed below.

T=P/(S+P)

$$CT=P_{1}/(S+P)$$

$$CT^{'}=P_{1}/P$$

Here, *S* is the phytolith concentration of the humic horizon (Ahorizon) (10^3^ particles/g); *P* is the total phytolith concentration of the soil profile (including the current phytolith concentration of the B, E, and C (or R) horizons), which is transported from the surface layers, but excluding the phytolith concentration of the humic horizon (10^3^ particles/g); and *P*_1_ is the phytolith concentration of the Chorizon (10^3^ particles/g).

*T* is the total translocation rate of phytoliths in the soil profile, and it reflects the intensity of vertical translocation of phytoliths. As *T* increases, there is an increase in phytolith translocation from the surface humic horizon to the lower layers of the soil profile. The larger the *T* value, the weaker are the preservation of soil phytoliths. *T* < 18 indicates that the translocation rate of phytoliths is relatively low, and thus, the phytoliths are better preserved; by contrast, *T* > 30 indicates that phytoliths are poorly preserved in the soil, and 18 < *T* < 30 represents an intermediate translocation rate. *CT* is the phytolith transport rate to the Chorizon, i.e., the translocation distance of phytoliths within the soil profile is increased. *CT* < 4 indicates that the phytolith translocation rate of the Chorizon is relatively low and that the translocation distance of phytoliths within the soil profile is relatively low, whereas *CT* > 12 represents a greater translocation distance, and 4 < *CT* < 12 represents an intermediate translocation rate to the Chorizon. *CT*′ is the relative transport rate of phytoliths to the Chorizon.

**Figure 7:**
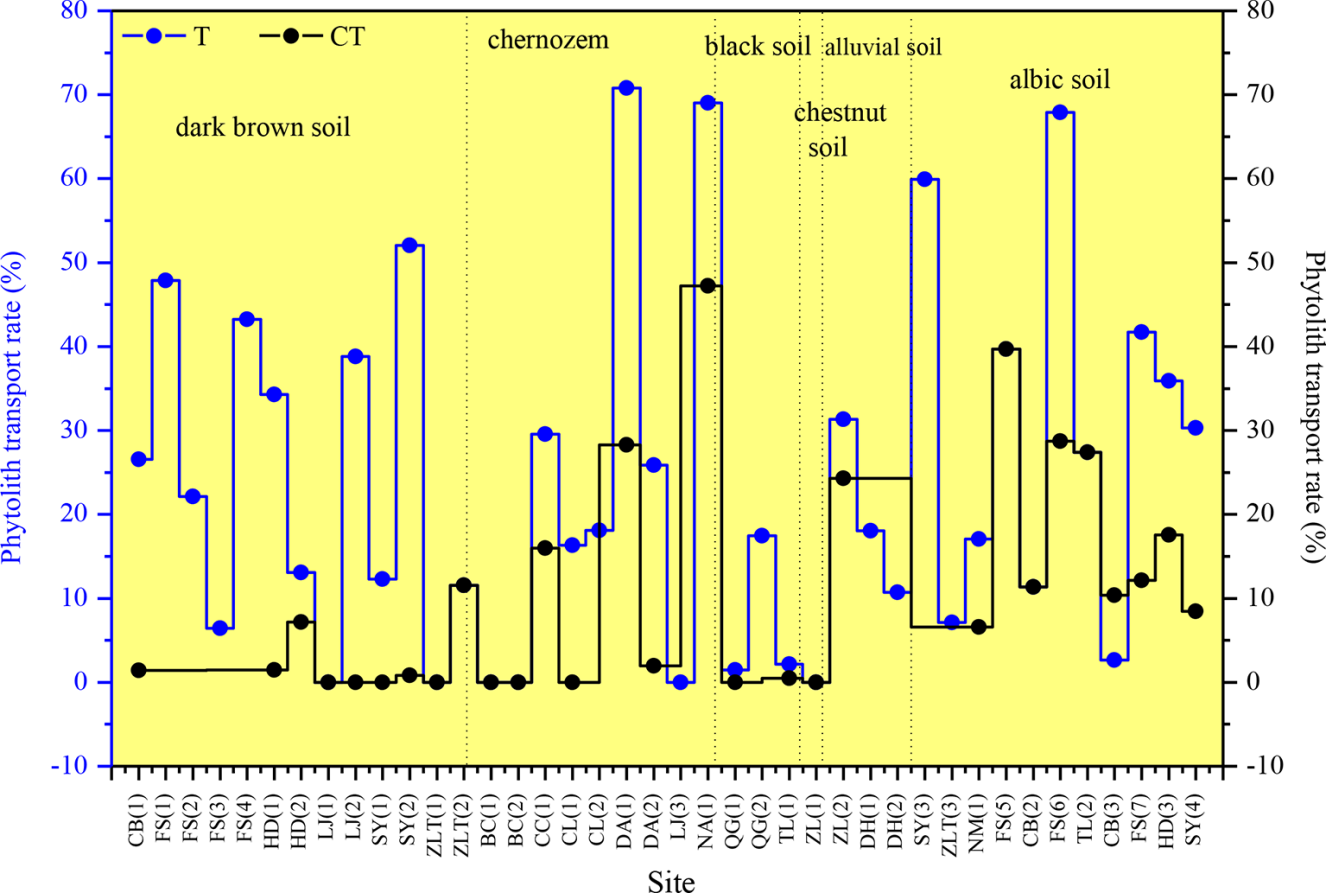
Total phytolith translocation rate (*T*) and phytolith translocation rate to the C horizon (*CT*) for soil profiles in Northeast China.

**Figure 8:**
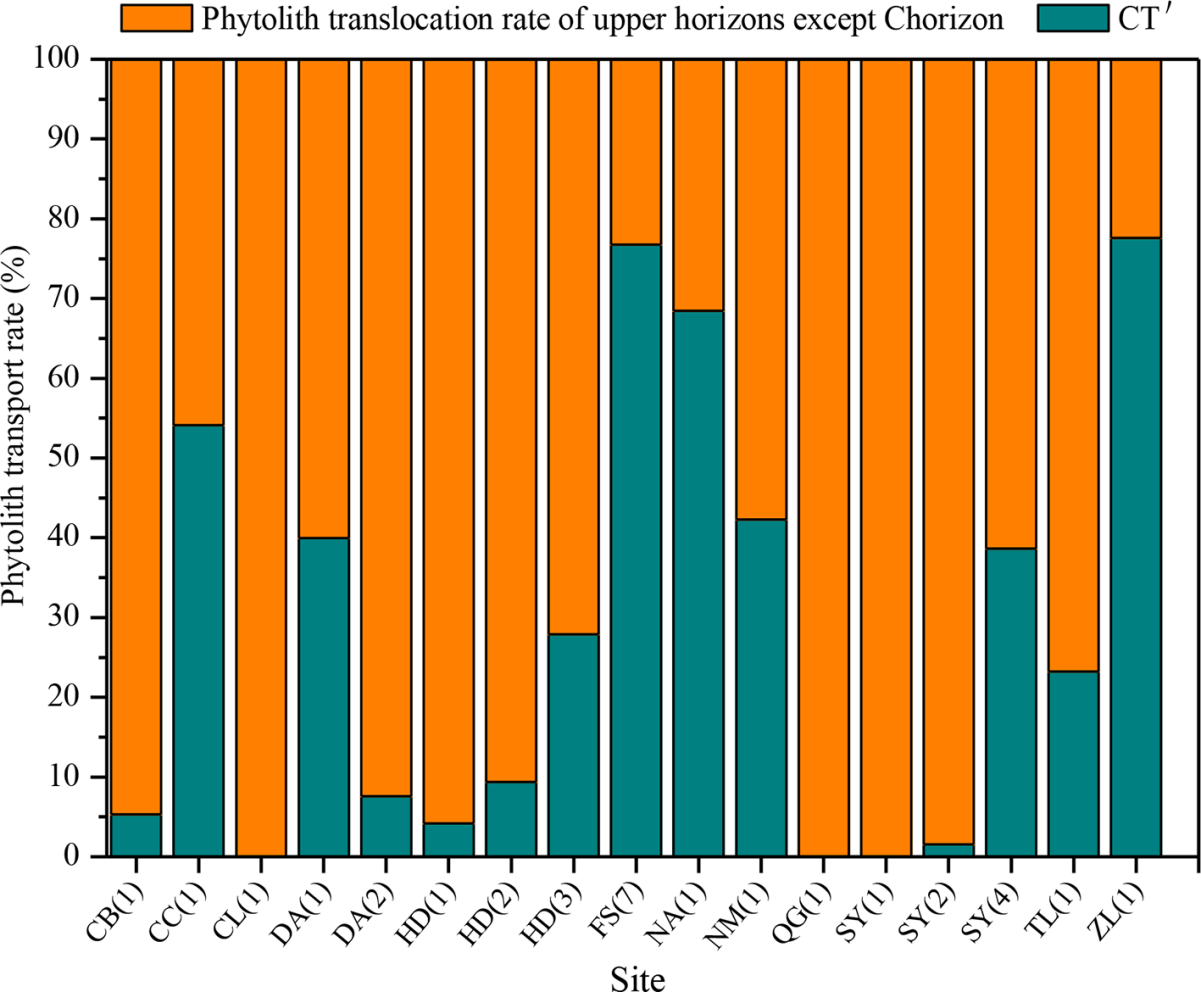
Relative translocation rate of phytoliths to the C horizon of (*CT*′) of soils in Northeast China.

There are substantial differences in *T* values among the various sampling sites in NE China (**Figure 7**). *T* ranges mainly from 0 to 40%, and the mean transport rate of phytoliths is 22%. In addition, *CT* is 10%. Specifically, the *T* values of total phytoliths for the chernozem and chestnut soils are lower (designated “low”), with values of 16 and 17%, respectively. *T* values of total phytoliths for dark brown soils and albic soils are greater (designated “intermediate”), with values of 22 and 28%, respectively. The *T* values of total phytoliths for black soils and alluvial soils are the largest (designated “high”), with values of 30 and 37%, respectively. Thus, the translocation rates of total phytoliths are lowest in chernozem and chestnut soils and highest in black soils and alluvial soils; whereas intermediate rates occurred in dark brown soil and albic soils. The distribution characteristics of the phytolith translocation rates among the different layers in the soil profiles are illustrated in **Figure 8**. On average, ∼28% of the total translocation rate of phytoliths occurs within the Chorizon of the soil profiles, i.e., ∼72% of the total translocation rate of phytolith occurs within the upper horizons. These findings demonstrate that translocation of phytoliths occurs in natural soils in NE China, but the transport distance is minor, and only a relatively small number of phytoliths are transported to the Chorizon.

#### Vertical Translocation Rates of Different Phytolith Types in Natural Soil Profiles

**Figure 9:**
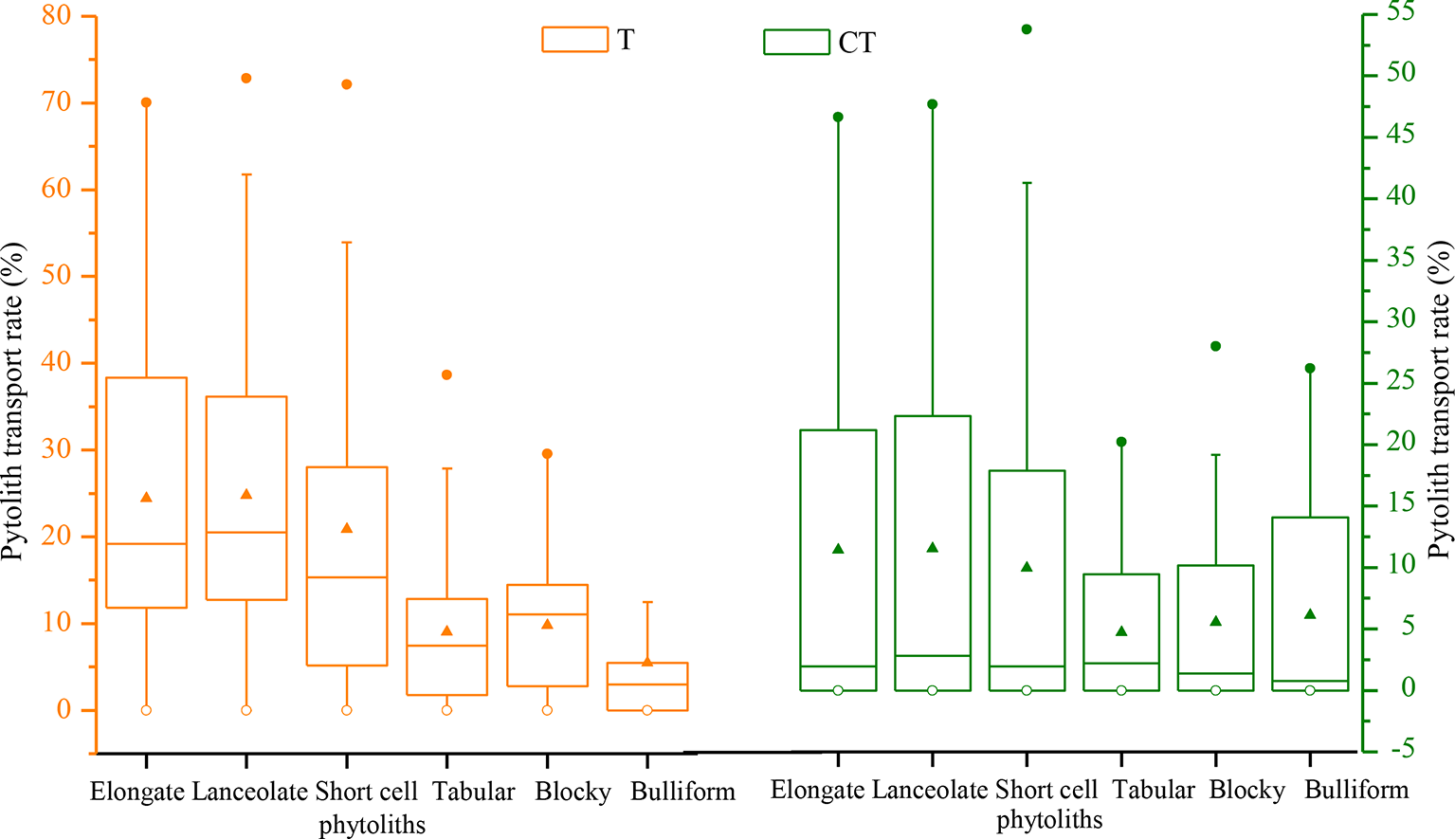
Box plots illustrating the distribution of total (*T*) and C horizon (*CT*) phytolith translocation rates for different phytolith morphotypes in soil profiles in Northeast China.

The translocation rates of the main phytolith types among the different sampling sites in NE China are illustrated in **Figure 9**. Evidently, there are substantial differences in translocation rate among the different phytolith morphotypes. In general, the translocation rates of short-cell, lanceolate, and elongate phytoliths are greater (designated “intermediate”), with respective rates of 21%, 25%, and 27%. The translocation rates of tabular, blocky, and bulliform phytoliths are lower (designated “low”), with respective rates of 18%, 16%, and 17%. The *CT* values of the main phytolith morphotypes also vary. Specifically, the *CTs* of lanceolate and elongate phytoliths are the largest (designated “high”), with respective rates of 14% and 13%; the *CT* values of short-cell, tabular, and blocky phytoliths range mainly from 4% to 12% (designated “intermediate”); and the *CT* of bulliform phytoliths is low (designated “low”), only 3%. These findings indicate that phytolith morphotype significantly affects the translocation behavior, with small phytoliths being translocated preferentially.

**Figure 10:**
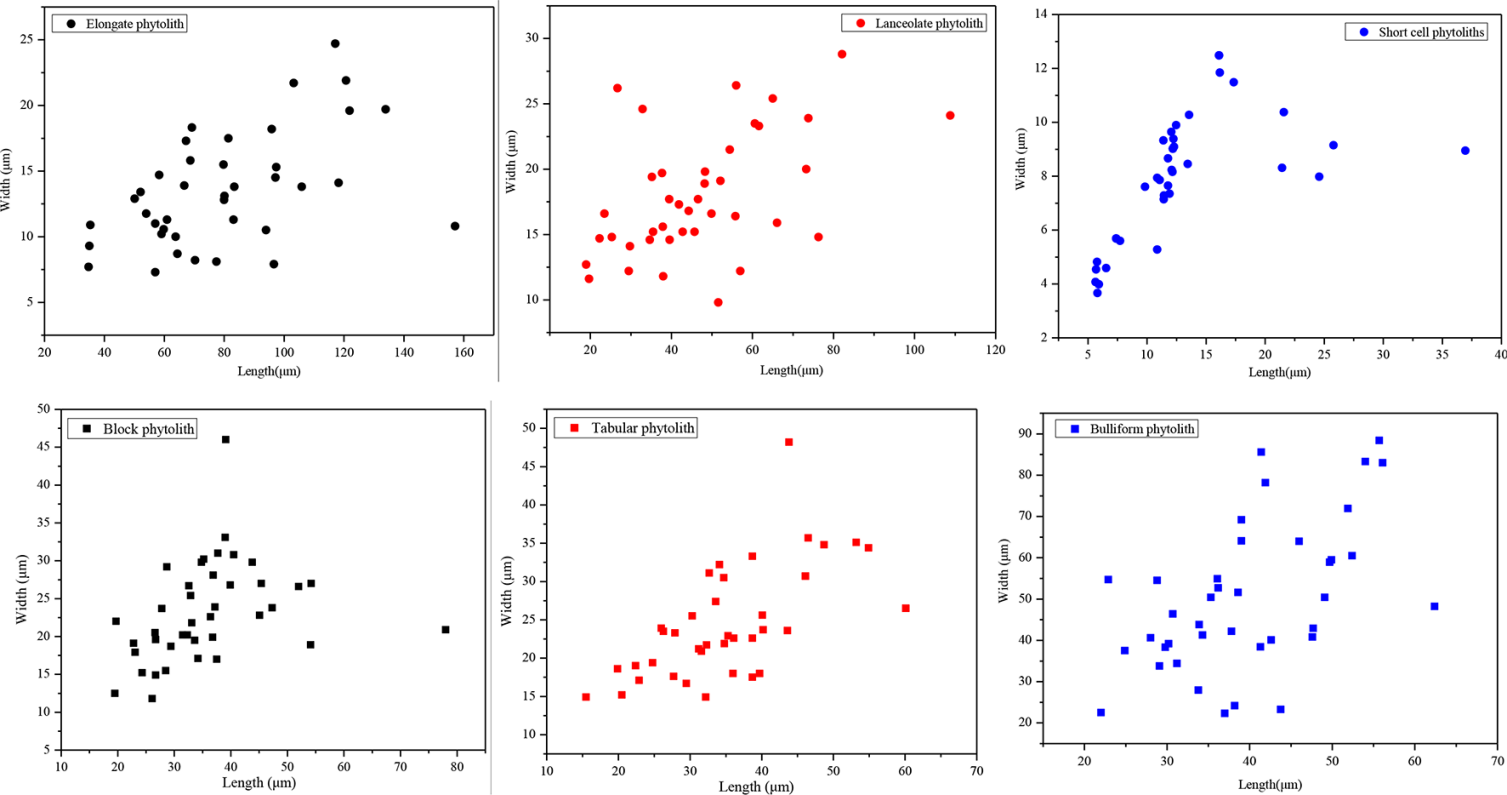
Scatter plots illustrating the relationship between width and length of different phytolith morphotypes in soil profiles in Northeast China.

In addition, we randomly measured the maximum length and width of short-cell, lanceolate, elongate, blocky, tabular, and bulliform phytoliths. For each phytolith type, 40 phytolith particles were measured. The maximum length and width of phytoliths were measured using the measuring tools provided by MOTIC software. The method used to determine the size parameters for different types of phytoliths is illustrated in Liu et al. (2016) and Gao et al. (2017). Scatter plots of the results are illustrated in **Figure 10**. For lanceolate and elongate phytoliths, their lengths are >30 μm, and their aspect ratios (namely, length/width ratio) are >2.5. The average length of short-cell phytoliths is 14 μm, which is smaller than that of the other phytolith types, and their average aspect ratio is 1.86. By contrast, the lengths of tabular, blocky, and bulliform phytoliths are mainly >25 μm, and their aspect ratios are 1.56, 1.53, and 0.79, respectively. Therefore, the soil phytoliths can be grouped into three categories according to their lengths, widths, and aspect ratios (**Table 3**). Combined with the results shown in **Figure 10**, it is evident that phytolith size and aspect ratio significantly affect their translocation behavior. Phytoliths with an aspect ratio >2 (e.g., lanceolate and elongate phytoliths) are all preferentially translocated, at rates mainly >18%, and the translocation rates of phytoliths with length <20 μm (e.g., short-cell phytoliths) are also mainly >18%, indicating preferential translocation. Specifically, phytoliths with length >30 μm and aspect ratio >2 and those with length <20 μm and aspect ratio <2 are preferentially translocated compared to those with length >25 μm and aspect ratio <2. Thus, it can be concluded that phytolith size and aspect ratio have a significant effect on phytolith translocation and that these attributes should be considered in future research on phytolith translocation.

**Table 3:** Definition of size and aspect ratio in soil profiles in Northeast China.

Phytolith type	Length (μm)			Width (μm)			Aspect ratio
	Maximum	Minimum	Mean	Maximum	Minimum	Mean	
Elongate	157.10	34.70	78.74	24.70	7.30	13.39	5.90 ( >2)
Lanceolate	108.80	19.00	47.23	28.80	9.80	17.97	2.63 ( >2)
Short cell phytoliths	36.95	5.64	12.73	12.48	3.67	7.87	1.62 ( <2)
Block	78.00	15.20	34.40	45.70	9.10	22.40	1.53 ( <2)
Tabular	65.10	15.50	33.70	48.00	10.50	23.30	1.45 ( <2)
Bulliform	62.40	22.00	39.57	88.40	22.30	50.01	0.79 ( <2)

## Discussion

### Phytolith Translocation Phenomenon in a Natural Soil Profile

Several researchers have suggested that phytolith translocation within soil profiles should be considered in paleoenvironmental reconstruction (Alexandre et al., 1999; Humphreys et al., 2003), whereas others have regarded phytoliths to be immobile (Rovner, 1983). Alexandre et al. (1999) reported phytolith translocation to a depth of 2.2 m in a ferrallitic soil, with a minor accumulation of phytoliths above an impermeable clay layer at the depth of 1.3–1.4 m. Humphreys et al. (2003) attributed the distribution of phytoliths in podzolic soils mainly to their translocation by percolating water; however, by contrast, Rovner (1983) concluded that phytolith mobility could be regarded as negligible for the purpose of paleoenvironmental reconstructions, due to their weight and large size. Piperno (2006) pointed out that the magnitude of translocation was probably minimal because phytoliths typically occurred only in the upper part of recent soils and their concentration usually decreased in the B horizon. However, in recent years, it has been found that due to various taphonomic events, soil phytoliths could be translocated from the soil surface, resulting in differences in phytolith content from the surface to the deeper horizons of soil profiles (Wallis, 2001; Farmer et al., 2005; He and Zhang, 2010; Golyeva and Svirida, 2017). Denis (2017) observed a relative increase in the concentration of phytoliths in the E horizon (at the depth of 25–30 cm) of an eluvial soil within a catenary sequence under field conditions, compared with the E and A′ horizons at the same depths, which confirms the occurrence of the downward translocation of phytoliths. The results of the present study confirm the occurrence of vertical translocation of phytoliths in natural soil profiles, which is consistent with the results of previous studies. In our study, the depth distribution of phytoliths in most profiles exhibits a similar pattern, with highest concentrations occurring in the humic horizon. However, there are several exceptions: at some sites, for example, the phytolith distribution exhibits the opposite distribution. This may be a result of the combination of soil type and the climatic conditions of NE China. Winter arrives early in NE China, and the interval of freezing is long, which greatly inhibits soil biological activity. As a result, organic matter produced within a growing season is not decomposed completely, which results in the accumulation of organic matter and the formation of a thick humic horizon. Consequently, the organic matter content of the gleyed horizon is increased. Several other studies have also reported that soil phytolith concentrations were closely related to soil organic matter content (Zhang et al., 2011). For Shuangyang (4) and Fusong (7), their soil types are albic soil. Studies have also reported that the gleyed horizon (E) of albic soils is always dominated by SiO_2_ particles and is firmer and contains a low porosity, which would be expected to result in only a limited movement of phytoliths to the next horizon (Yan et al., 2018). For Shuangyang (3), it belongs to black soils. The degree of humification of the illuvial horizon of this soil was higher than that in the other soil types; to a certain extent, organic matter can absorb and polymerize phytoliths, resulting in the humified layer having high phytolith content. Accordingly, the phytolith concentration of the gleyed horizon at these sites is increased.

In recent years, phytolith translocation studies based on experiments (e.g., irrigation, a fluorescent labeling technique) have further confirmed the occurrence of phytolith translocation in soils (Fishkis et al., 2009; Fishkis et al., 2010a; Fishkis et al., 2010b). However, current research on the vertical translocation of phytoliths in soils is based mainly on experiments, which often overlook the complexity of factors influencing the vertical translocation of soil phytoliths under natural conditions. Consequently, this prevents a full understanding of the postdepositional processes affecting phytoliths in soils. Thus, the vertical translocation of soil phytoliths in natural soil profile should be assessed, and phytolith translocation rates in a natural soil profile should be confirmed.

In the primary stage of soil formation, soil material mainly consists of lithophytes such as lichen and moss. Phytoliths are particles of hydrated silica (SiO_2_•*n*H_2_O) of phytogenic origin present in the tissues of many vascular plants or bryophytes, and they are typically deposited in plant cells or in the intercellular spaces of plants (Piperno, 2006). In addition, phytolith fragments have been observed in bryophytes, but morphogenetic phytoliths do not exist in bryophytes (Piperno, 2006). In the primary stage of soil formation, morphogenetic phytoliths do not exist in the soil; that is say, if phytoliths do occur, their morphology should be markedly different from those observed so far because the phytolith morphologies observed so far have a constant species source. Therefore, the C horizons of natural soil profiles developed on bedrock and consisting of weathering products may not contain phytoliths, and any phytoliths present are possibly derived from phytolith translocation from the upper soil layers.

*Soil organic matter content*. Soil organic matter is the carbon-containing component in soil and consists of residues of various plants and animals, soil microorganisms, and decomposed and synthesizes substances. When the parent plants die and decay, the phytoliths are preserved in soils and sediments on timescales of up to millions of years. Thus, soil phytolith concentration is intimately related to soil organic matter, and previous research has shown that soil phytoliths are closely related to soil organic matter (Zhang et al., 2011). Generally, the predicted phytolith concentrations of the illuvial (B) and eluvial (E) horizons of the soil profiles are all significantly lower than the original values. It is likely that, over time, soil phytoliths are dissolved, broken, and lost due to various taphonomic processes, and therefore, the phytoliths of the soil profiles derived from the aboveground vegetation predicted by the regression relationship are larger than the actual values. Thus, we suggest that, when a soil horizon develops within a profile, the phytolith concentration derived from the aboveground vegetation is substantially less than the current measured value in that horizon. Hence, for a given soil horizon, its excess phytoliths are possibly caused by the translocation of phytoliths from the upper layers, rather than supplied from the aboveground vegetation, although its source is not the only translocation process. This suggests that the translocation of phytoliths possibly occurs in natural soils.

*Soil pH*. Soil pH is an additional factor affecting phytolith preservation. Soil pH varies with soil type, depth, and horizonation. In the studied soil profiles, pH increases with depth (**Figure 4**). The pH values range mainly from 3 to 9, and only a few sites have pH values exceeding 9. Several studies have indicated that phytoliths are well preserved within the soil pH range of 3–9, whereas when soil pH exceeds 9, they are readily dissolved (Bremond et al., 2017). In addition, it has been found that, when soil pH exceeded 8, there was an increase in the number of phytoliths affected by dissolution (Fraysse et al., 2006; Karkanas, 2010). Thus, soil phytoliths are poorly preserved under alkaline pH conditions, and hence, the pH of the lower layers within a soil profile inhibits phytolith preservation or results in their complete dissolution. In our study, in the lower soil layers, soil pH is high, whereas the content of poorly preserved phytoliths is also high. This trend is consistent with the influence of soil pH on phytolith preservation. The preservation of soil phytoliths is likely influenced by numerous factors, and the mechanisms involved are poorly understood. In the present study, soil pH is likely a major factor affecting phytolith preservation (see also Li et al., 2005; Fraysse et al., 2006). Hence, we infer that the high content of poorly preserved phytoliths in the lower layers of some studied soil profiles is at least partly the result of phytolith translocation.

*Phytolith size*. It has been suggested that the downward movement of pollen in soils results from the downward percolation of surface water and that if the process occurs to a significant extent, pollen grains will be separated by size, with the concentration of small pollen grains increasing with depth (Walch et al., 1970). By analogy, if there is substantial phytolith translocation within a soil profile, the content of small phytoliths should also increase with depth. In the present dataset, the size distribution of phytoliths with depth within the profiles is consistent with this inference. The content of small phytoliths increases with depth, whereas the content of large phytoliths decreases. Thus, we conclude that, in natural soil profiles, phytoliths may be translocated from the upper to lower layers.

In conclusion, potential translocation exists in soil phytoliths, and the translocation bias of soil phytoliths is a concern for deep-time studies, as this would improve their accuracy with respect to phytolith assemblage reflecting original ecosystem types. However, more investigations are needed to further understand how soil phytoliths translocate to lower layers of soil profile before conducting a phytolith-based paleovegetation reconstruction.

### Phytolith Translocation Rates in a Natural Soil Profile

An experimental study of the translocation rates of phytoliths in loamy and sandy soils confirmed this phenomenon (Fishkis et al., 2009, Fishkis et al., 2010a; Fishkis et al., 2010b). Fishkis et al. (2009) investigated phytolith translocation in sandy sediments under different rainfall conditions and found that, under high-frequency irrigation, 22% of the applied phytoliths were removed from the application layer. In addition, the results of the present study demonstrate that ∼22% of the phytoliths were transported below the surface of natural soils in NE China. The phytolith translocation rates observed in our study are consistent with the results of other studies of experimental studies of phytolith translocation within soils.

We also observe differences in the vertical translocation rate of phytoliths among the studied soil types. The total translocation rates in chernozem and chestnut soils, and to a lesser extent in dark brown soil and albic soils, are all significantly smaller than in black soils and alluvial soils. In dark brown soils, they have a loose and porous structure, which is conducive to the vertical translocation of phytoliths, while the stronger earthworm activity in the humus horizon of dark brown soils may enhance the leaching of phytoliths. For albic soils, studies have shown that the clay particles in albic soils can be transported downwards with percolating water (Xiu et al., 2019). Notably, we found that the content of clay particles in the B layer and lower layers of albic soils was higher than in the upper layer (**Table 4**). Therefore, mechanical leaching occurs in these soils, namely the displacement of clay particles, which is consistent with the results of previous studies (Institute of Forestry Soil, Chinese Academy of Sciences, 1980). Moreover, soil clay particles can adsorb silicic acid; within a specific pH range, as the soil pH and the content of soil clay particles increase, the adsorption of both clay particles and silicic acid also increases (Zhang and Zhang, 1996). Therefore, with the mechanical leaching of clay particles in albic soils, the vertical translocation rate of phytoliths may also be relatively high. For alluvial soils, these soils are coarse textured, containing gravel particles with large interstices (**Table 4**). In addition, the sampling sites of alluvial soils are mainly located in the eastern mountainous and forested region of Northeast China, where erosion by rainfall and flowing water is strong. These conditions favor a high translocation rate of phytoliths. Conclusively, these findings all demonstrate that soil type is an important factor in determining phytolith translocation rates.

**Table 4:** Textural composition of albic and alluvial soils.

Soil type	Site	Soil genetic layer	Sand/%	Silt/%	Clay/%
Albic	Changbai(3)	A-horizon	39.62	54.51	5.87
		E-horizon	0.57	86.44	12.99
		B-horizon	67.81	29.21	2.98
	Fusong(7)	A-horizon	12.73	78.31	8.96
		E-horizon	2.56	81.97	15.47
		B-horizon	23.18	72.47	4.35
	Huadian(3)	A-horizon	87.02	12.98	0
		E-horizon	2.56	81.97	15.47
		C-horizon	39.92	54.31	5.77
Alluvial	Fusong(5)	A-horizon	59.77	36.45	3.78
		C-horizon	89.56	10.44	0
	Changbai(2)	A-horizon	80.27	19.73	0
		C-horizon	82.82	17.18	0
	Fusong(6)	A-horizon	39.02	57.68	3.3
		B-horizon	0	96.51	3.49
		C-horizon	22.72	72.22	5.06

A, humus horizon (Ahorizon); B, illuvial horizon (Bhorizon); C, parent rock horizon (Chorizon); E, eluvial horizon (Ehorizon).

### Effect of Phytolith Size on Its Translocation

Research on the vertical translocation of different phytolith types has been carried out in several geographical regions, and it has concentrated on an experimental approach (Fishkis et al., 2009; Fishkis et al., 2010a; Fishkis et al., 2010b). In these studies, plant phytoliths were added to sandy sediment (free of phytoliths) or other soil types, and changes in phytolith concentration with depth were observed to determine the translocation rates of different phytolith types. However, soil phytoliths are derived from a wide variety of plant species (including both herbaceous plants and trees), and therefore, the limited number of phytolith morphotypes used in experimental studies does not enable a comprehensive analysis of the factors affecting phytolith translocation. Our study of the translocation rates of different phytolith morphotypes in natural soils has revealed contrasts in translocation rates among different phytolith types, with smaller phytoliths being preferentially displaced. Previous studies have also shown that phytolith shape (such as length/width ratio) had a significant effect on translocation, with small phytoliths being most affected (Locke, 1986; Fishkis et al., 2010a). Our results are also consistent with those of column experiments on soil colloids, microorganism, and biochar in packed sand or soil (Weiss et al., 1995). Gannon et al. (1991) observed the more rapid translocation of small microorganisms in soil columns compared with large microorganisms. In addition, Zhang et al. (2010) reported that coarse biochar was readily deposited during mechanical filtration, whereas fine biochar was preferentially displaced. Similarly, Sun et al. (2012) reported that, in the same soil, there was a stronger surface adsorption effect between large soil colloids (2,049.9 nm) and the rest of the soil, compared to that observed for small soil colloids (246.15 nm), and the effect of this phenomenon was to reduce the movement of large soil colloids. As in the case of soil colloids, microorganisms, and biochar, phytolith size has a significant effect on translocation, with phytoliths of smaller diameter being preferentially translocated. Overall, our results, together with those of previous studies, emphasize the need to consider the effects of differential phytolith translocation in studies which attempt to use soil phytoliths for paleoenvironmental reconstruction.

Our results also demonstrate that aspect ratio has a significant effect on phytolith translocation: phytoliths with length >30 μm, aspect ratio >2 and those with length <20 μm and aspect ratio <2 are preferentially translocated compared to those with length >25 μm and aspect ratio <2. These findings contrast with those of previous studies. For example, Weiss et al. (1995) reported a higher fraction of round bacteria in effluent passing through a packed sand column compared to the inflowing suspension. Similarly, Salerno et al. (2006) observed the more rapid translocation of rounded polystyrene latex particles compared to elongated particles in columns composed of glass beads. We suggest that the discrepancies between our results and these other studies reflect the different transport mechanisms of bacteria or soil colloids compared to phytoliths. Whereas the transport of bacteria or soil colloids is mainly controlled by diffusion and surface interactions, that of phytoliths latter is strongly affected by hydrodynamic shear and mechanical capture in small pores (Bradford and Bettaha, 2005, Bradford et al., 2006; Foppen and Schijven, 2006). Hence, the preferential transport of circular bacteria or colloids could be attributed to the smaller specific surface of rounded versus elongated particles, whereas the preferential transport of elongated phytoliths maybe due to the higher probability of detachment by water flowing through different pores. In addition, the results of correlation analysis of phytolith translocation rate and soil clay content indicate that the relationship between translocation rates of different phytolith types and soil clay content varies: translocation rates of phytoliths with length >30 μm and aspect ratio >2 and with length <20 μm and aspect ratio <2 are significantly positively correlated with soil clay content (*p *< 0.05). In contrast, the translocation rates of phytoliths with length >25 μm and aspect ratio <2 are negatively correlated with soil clay content (*p* >0.05) (**Table 5**). Our study provides direct evidence for a close relationship between phytolith translocation rate and soil clay content, which indicates the preferential adsorption of elongated phytoliths and small phytoliths by soil clay particles. In other words, when clay particles are translocated within the soil profile, the translocation rate of elongated phytoliths and small phytoliths is increased. The pronounced differences in total translocation rates among different phytolith types results in differences in the phytolith characteristics of soil horizons. Hence, differences in the percentages of different phytolith types with depth within a soil may reflect not only changes in vegetation but may also reflect the differential translocation of different morphotypes. This effect clearly needs to be considered in paleoenvironmental studies using phytoliths.

**Table 5:** Correlation coefficients for the relationship between the *T* value of phytoliths and clay content of soil profiles in Northeast China.

	Clay (%)	Phytoliths with length >30 μm and aspect ratio >2	Phytoliths with length <20 μm and aspect ratio <2	Phytoliths with length >25 μm and aspect ratio <2
Clay (%)	1			
Phytoliths with length >30 μm and aspect ratio >2	0.801*	1		
Phytoliths with length <20 μm and aspect ratio <2	0.848*	0.69	1	
Phytoliths with length >25 μm and aspect ratio <2	−0.764	−0.572	−0.36	1

*Correlation is significant at the 0.05 level (two-tailed).

## Conclusion

1. In most of the studied soil profiles in NE China, the depth distribution of phytoliths exhibits a similar pattern, which demonstrates the preferential accumulation of phytoliths within the humic horizon. Therefore, differences in phytolith concentration as a function of depth should be considered when interpreting soil phytolith assemblages for paleoclimate and paleovegetation reconstruction.
2. Through the relationship between the phytolith concentration and organic matter content of soil surface horizons, together with observations of the distribution characteristics of phytoliths based on size and preservation, we conclude that phytoliths are transported below the surface of natural soils in NE China. We estimate that ∼22% of total phytoliths are translocated below the surface of natural soils, although the translocation distance is limited.
3. There are substantial differences in the total translocation rate among different phytolith types, with phytolith size and aspect ratio having a significant effect: phytoliths with length >30 μm and aspect ratio >2 and those with length <20 μm and aspect ratio <2 are preferentially translocated compared to those with length >25 μm and aspect ratio <2. These results indicate that phytolith size and aspect ratio should be considered in future studies of phytolith translocation.

## Data Availability Statement

All datasets generated for this study are included in the manuscript/supplementary files.

## Author Contributions

DJ designed the study. LL, HL, GG, DL, and NL organized field work. LL and HL carried out phytolith analysis. LL analyzed statistics and designed most figures, except one which was designed by NL. All authors helped to write and proofread the manuscript.

## Funding

This study was supported by the National Science Foundation of China (grants 41901097, 41971100, 41771214), the Natural Science Foundation of Hunan Province, China (grant, 2019JJ50371), Scientific Research Project of Education Department of Hunan Province, China (grant 18B029), and the Construct Program of the First-class Discipline (geographic science) in Hunan Province, China.

## Conflict of Interest

The authors declare that the research was conducted in the absence of any commercial or financial relationships that could be construed as a potential conflict of interest.
